# Analysis of Coupled Response Characteristics of NAI Release and Stem Flow in Four Urban Greening Tree Species in Beijing During Drought Stress and Recovery Processes

**DOI:** 10.3390/plants14172630

**Published:** 2025-08-23

**Authors:** Xueqiang Liu, Bin Li, Weikang Zhang, Shaowei Lu, Jigui Wu, Jing An, Yaqian Fan, Na Zhao, Xiaotian Xu, Shaoning Li

**Affiliations:** 1 Institute of Forestry and Pomology, Beijing Academy of Agriculture and Forestry Sciences, Beijing 100093, China; 13925813899@163.com (X.L.); libin@baafs.net.cn (B.L.); lushaowei@baafs.net.cn (S.L.); xuxiaotian@baafs.net.cn (X.X.); 2 College of Forestry, Shenyang Agricultural University, Shenyang 110866, China; zhwk1986@syau.edu.cn; 3 Beijing Yanshan Forest Ecosystem Research Station, National Forest and Grassland Administration, Beijing 100093, China; 4 Beijing Songshan Natural Reserve Administration, Beijing 102115, China; 13616009329@163.com (J.W.); annsilence@126.com (J.A.); fyq1018@126.com (Y.F.)

**Keywords:** negative air ions, sap flow, greening tree species, drought stress, open-top chamber (OTC)

## Abstract

Negative air ions (NAI) represent an important ecological value indicator for green tree species. Flow of sap is a crucial indicator for water utilization and physiological state of trees. Although there have been some advancements in studies on the correlation between the release of NAI by plants and sap flow in recent years, it is still unclear how the release of NAI by plants changes during drought stress and recovery processes, as well as the coupling effect between the release of NAI by plants and sap flow under drought stress. In this context, four typical green tree species, *Robinia pseudoacacia*, *Quercus variabilis*, *Pinus tabulaeformis*, and *Platycladus orientalis*, were selected as experimental materials. A drought stress and recovery control experiment was conducted based on OTC. The dynamic data of negative air ion concentration (NAIC) and sap flow rate during the process of drought stress and recovery were monitored to clarify the characteristics and correlations of NAI and sap flow changes in the experimental tree species under drought stress and recovery. The main research results are as follows: (1) At the end of the drought period, the NAI and sap flow in the drought treatment group significantly decreased (*p* < 0.01), compared with the control group (CK), and the reduction rate of sap flow (77.73 ± 4.96%) for each tree species was higher than that of NAI (47.78% ± 4.96%). (2) At 1 day after rehydration, the recovery amplitudes of NAI and sap flow for all tree species were the greatest; at 7 days after rehydration, the NAI and sap flow of the drought treatment group recovered to the levels of the control group (*p* > 0.05). (3) During different stages of drought rehydration, the response degree of NAI to sap flow varied. The study found that in the drought-rehydration stage, the correlation between the NAI released by each tree species and sap flow was the lowest at the drought endpoint. In conclusion, this research clarifies the changing patterns of plant NAI release and sap flow during drought-rehydration, as well as the response changes of NAI to sap flow. It provides a theoretical basis for selecting drought-tolerant tree species in arid regions.

## 1. Introduction

Water is the main factor influencing the distribution of plant species and the structure, function, and productivity of ecosystems [1,2]. Water shortage is the most common abiotic stress that restricts plant growth in arid and semi-arid regions. Severe limitation of water utilization by plants can lead to retarded plant development, reduced dry weight of plant organs, and even plant death [3]. Although some woody plants can recover from severe droughts [4], widespread decline events of forest landscapes occur frequently worldwide [5,6].

The generation of NAI mainly includes natural generation and artificial generation. The methods for preparing NAI in nature include lightning strikes, waterfall effects, and the release by plants, etc. The production of NAI by plants mainly occurs through tip discharge, photoelectric effect, and photosynthetic physiological processes [7,8]. The research has determined that NAI consists of various ions, including O^−^, O^2−^, O^3−^, CO^3−^, CO^4−^, HCO^3−^, NO^2−^, NO^2−^, etc. Moreover, the NAI released by plants will combine with water molecules produced by plant transpiration to form ion clusters such as O^2−^(H_2_O)_n_ and CO^3−^(H_2_O)_n_, thereby prolonging the survival time of NAI [9,10]; Further studies have shown that indicators including plant sap flow, which can reflect the plant’s transpiration capacity, significantly affect the plant’s ability to produce NAI [11]. In arid regions, plants release NAI. Due to the impact of arid conditions on the physiological indicators of plants and the relatively low air humidity, the level of NAI produced by plants in arid regions is lower than the normal level [12,13]. However, the current research mainly focuses on the situation under natural conditions, where the release of NAI by plants is affected by drought in arid regions. There is a lack of studies on the impact of drought stress on the release of NAI by plants under controlled conditions.

Sap flow is an important part of water transport within plants, and water transport within plants involves many processes such as water balance, photosynthesis, transpiration, and carbon cycle within the plant, and even limits other physiological processes of the plant [14,15]. Studies have shown that in arid regions, the sap flow of plants undergoes significant changes, and even the water distribution within the plants changes [16]. Additionally, research has revealed that in summer, a decrease in soil moisture leads to a reduction in the sap flow of plants [17].

Relevant studies have shown that drought can have an impact on the physiological processes of plants, especially having a significant effect on the plant’s sap flow. However, it is still uncertain how the changes in NAI release by plants occur during the drought stress and recovery processes, and the coupling effect between NAI release by plants and sap flow during the drought stress and recovery processes remains unclear. We therefore hypothesized that during the process of drought stress and post-drought recovery, NAI and tree stem sap flow will undergo significant changes. This study monitored the dynamic data of NAI and sap flow released by plants by conducting drought stress and recovery control experiments, and evaluated the changes in the correlation between the release of NAI and sap flow by different greening tree species during the drought stress and recovery processes. This research can provide data support and a theoretical basis for the selection of green tree species and irrigation management in arid and semi-arid regions.

### 1.1. Overview of the Research Area

The research site is located within the Forestry and Fruit Tree Research Institute of Beijing Academy of Agriculture and Forestry Sciences (39°58′ N 116°13′ E). This site is situated at the foot of Xiangshan Mountain, with an altitude of approximately 88 m, and covers a total area of 13.33 hectares. Beijing is located in a warm temperate, semi-humid continental monsoon climate zone. It has distinct seasons, with shorter springs and summers and longer winters and autumns; summers are hot and rainy, while winters are cold and dry. The annual average temperature ranges from 11.0 °C to 14.2 °C, and precipitation mainly occurs in summer (July to August) [18].

### 1.2. Selection of Experimental Tree Species

The test tree species were selected based on the species that are widely distributed and representative in Beijing and are also among the recommended afforestation plants for the Beijing Plain Afforestation Project (2014 revision). This included two conifer species: *Pinus tabuliformis* and *Platycladus orientalis*, and the two deciduous broad-leaved tree species *Robinia pseudoacacia* and *Quercus variabilis*. The growth status of each species is shown in Table 1. The tree species used were all in good growth condition, with uniform shapes and no diseases or pests. They were 3-year-old seedlings. The seedlings were planted in flower pots with a height of 37 cm, an upper diameter of 37 cm, and a lower diameter of 30 cm. The substrate in the pot was the seedling substrate, and the ratio of the substrate in the pot is shown in Table 2.

### 1.3. Drought Treatment and Experimental Design

Tree species selection: For this experiment, 12 test trees of the same species with good growth conditions and consistent growth were selected for use as test trees. They were randomly divided into 2 groups, with the drought rehydration treatment group and the normal irrigation control group (CK) treatment set up respectively. Each group contained 6 trees, and the volume of soil moisture in the pots was monitored to ensure the accuracy of the NAI data. A blank control group without plants was also set up to ensure the accuracy of the monitoring. Monitoring nodes: Before the treatment, at the drought endpoint, 1 day after rehydration, 3 days after rehydration, and 7 days after rehydration. The soil volumetric water content (SWC) was monitored using Time Domain Reflectometry (TDR). The SWC at the wilting point for each tree species was as follows: *P. tabuliformis* (9.3%), *P. orientalis* (10.6%), *R. pseudoacacia* (12.7%), and *Q. variabilis* (11.4%).

Drought rehydration treatment: For each tree species, 6 plants were selected, and water was given until the soil volume water content reached 80–90% of the field capacity. Then, the watering was stopped to allow the young trees to experience natural gradual drought until the soil moisture content reached the wilting point of the tree species, completing the drought stage. Then, rehydration was carried out to maintain the soil volume water content at 90% of the field capacity in the pots.

Control treatment: For each tree species, 6 pot-grown young seedlings were selected as the control group. During the entire experiment, they were all watered, and the soil volume water content was monitored in real time to keep it at 90% of the field capacity throughout the experiment.

Blank control group: The same number of pots with soil wrapped in polytetrafluoroethylene film were placed in the control group.

### 1.4. OTC Monitoring

**Figure 1:**
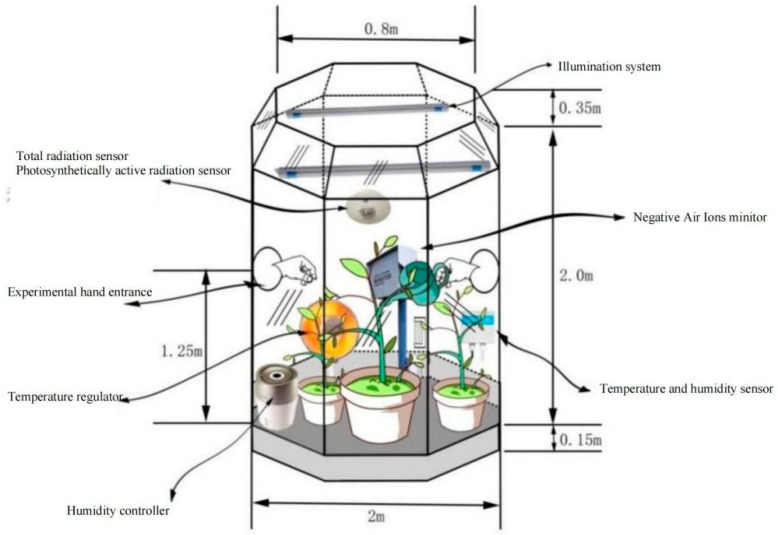
Schematic diagram of open-top chamber (OTC).

Before the experiment began, the dust on the leaf surfaces was removed using a sprayer, and the leaves were dried. Then, the leaf surfaces were wiped with a damp cloth. A polytetrafluoroethylene film was wrapped around the flower pots, and the plant heights and stem diameters were measured [11]. The natural evaporation of soil moisture and the occurrence of NAI in the soil were prevented to avoid interference with the experiment. During the drought experiment, based on the open-top chamber (OTC) environmental control system, the temperature, relative humidity, and light intensity inside the OTC were consistent with those outside. When at the monitoring node, the plants were placed in the OTC, and the NAI and sap flow data were continuously monitored for 24 h. The specific shape and principle of OTC are shown in Figure 1.

### 1.5. Data Acquisition

#### 1.5.1. Monitoring of NAI Concentration

Continuous monitoring of NAI concentration used the Yconetest-500 (Beijing Lanruxin Technology, model: AQ3600, Beijing, China) negative ion monitoring system. It is equipped with a remote monitoring template internally and transmits NAI data to the cloud. This negative ion monitoring system has a measurement range of 0–5.0 × 10^6^ ions·cm^−3^, measures small-sized NAI with an ion mobility of ≥0.7 cm^2^/(V·s), and has an ion concentration measurement error of ≤5%. The collection interval was set at 10 min. The value of the negative air ion concentration (NAIC) released by the plants in this study was

NAI_Plant_ = NAI_Treat_ − NAI_B_ (1)

In the formula, NAI_Treat_ represents the average value of NAIC in the open-top chamber with a plant experiment group during this period (ions·cm^−3^); NAI_B_ represents the average value of NAIC in the open-top chamber without a plant experiment group during this period (ions·cm^−3^).

#### 1.5.2. Detection of Sap Flow in Tree Trunks

A plant sap flow instrument in a wrapped form (Beijing Yugen Company, model: RR-BG10, Beijing, China) was used to wrap the tree trunk, and a constant voltage was connected. A CR1000 data collector was connected for data collection. The measurement interval was set at 15 min. The wrapped plant sap flow instrument utilizes the principle of energy balance, that is, the total heat input Q, the heat Q level transferred to the external environment, the vertical upward heat Q transmitted up through the stem, and the vertical downward heat Q transmitted down through the stem, which are measured to calculate the heat Q_f_ carried away by the stem flow, and then, the stem flow sap flow rate of the plant is calculated.

Q = V^2^/R (2)

In the formula, Q represents the total heat (W); V represents the heating voltage (V); and R represents the heating resistance (Ω).

Q_v_ = K_st_·A·(T_BH_ − T_AH_)/dX (3)

In the formula, Q_v_ represents the heat transferred to the external environment (W); K_st_ is the heat conduction rate of the stem (W/(m·K)); A is the cross-sectional area of the stem (m^2^); T_BH_ is the temperature difference between point B and point Hb (°C); T_AH_ is the temperature difference between point A and point Ha (°C), and dX is the distance between the two thermocouple TC nodes (m).

K_sh_ = (Q − Q_v_)/(T_CH_·0.04) (4)

In the formula, K_sh_ represents the thermal conductivity constant (W/m·V); T_CH_ is the temperature difference between point C and point Hc (°C).

Qr = K_sh_·(T_CH_·0.04) (5)

Note: K_sh_ represents the average value for the period before sunrise.

Qf = Q – Q_r_ − Q_v_ (6)

In the formula, Q_f_ represents the heat carried away by the stem flow (W).

dT = (T_BH_ + T_AH_)/2 (7)

In the formula, dT represents the temperature difference of the stem flow (°C).

F = Q_f_/(Cp·dT)·3600 (8)

In the formula, F represents the sap flow rate (g/h); Cp (the heat capacity of water) = 4.186 J/(g·°C).

### 1.6. Data Statistics

Based on the NAIC data and sap flow data collected by the NAI automatic detection system and the sap flow monitoring system, the basic data processing of NAI and sap flow data was carried out using Excel 2019 (Microsoft, Redmond, WA, USA). Regression analysis and Pearson correlation analysis of NAI and sap flow were conducted using SPSS Statistics 26 software (IBM, Inc., Armonk, NY, USA). The statistical significance was set at *p* < 0.05 for two-tailed test. The significant differences between the drought treatment group D and the control group CK were analyzed using the Independent Samples *t*-test. The relationship chart between NAI and sap flow rate after analysis and processing was drawn using Origin 2024 (OriginLab, Northampton, MA, USA). NAI data processing: After the experiment, the obtained NAI data were processed according to the “Observation Technical Specification for Air Negative (Positive) Ion Concentration” (LY/T 2586—2016) issued by the National Forestry Administration in 2016. The obtained NAI data were screened and sorted using an Excel table. The specific screening methods are as follows: (1) Abnormal data caused by instrument failure were discarded; (2) Multiple consecutive identical values were discarded; (3) Values differing by three times or less than 1/3 were discarded; (4) Missing values were filled using interpolation.

## 2. Results and Analysis

### 2.1. Characteristics of NAI Release and Sap Flow Changes in Different Tree Species During Drought Stress Process

**Figure 2:**
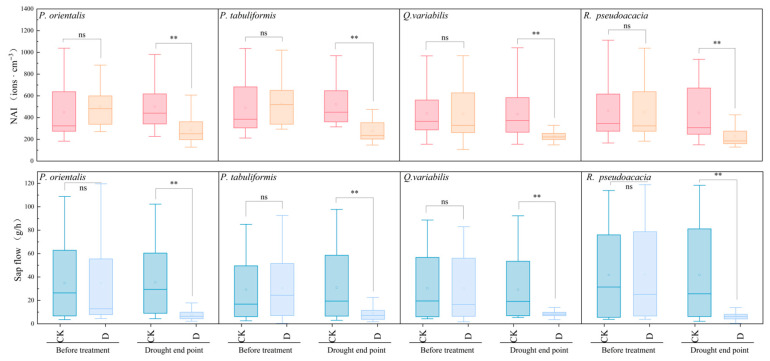
Characteristics of NAI release and sap flow of tree species in drought stage. “**” indicate significant differences among groups (** *p* < 0.01), “ns” indicates no statistical difference.

Based on the open-top chamber (OTC) drought experiment as shown in Figure 2, during the drought stress process, the changes in NAI and sap flow released by the plants under drought treatment were the same, and both decreased significantly (*p* < 0.01). Before the drought treatment, the daily average NAI of the drought treatment group (D) was 477.63 ± 36.94 ions·cm^−3^, and the daily average sap flow was 34.47 ± 4.92 g/h. As the soil volumetric water content of each tree species in the drought treatment group D decreased, the plants suffered from drought stress, and both the NAI release capacity and sap flow of the plants decreased. At the end of the drought, the daily average NAI of the D group was 253.75 ± 26.4 ions/cm^3^, and the daily average sap flow was 7.97 ± 1.02 g/h.

Compared with the control group (CK), the drought treatment group showed significant differences in both NAI and sap flow at the drought endpoint. However, the decline in NAI of coniferous trees at the drought endpoint was 45.72 ± 1.28%, while that of broad-leaved trees was 49.24 ± 2.02%. The decline in NAI of broad-leaved trees was greater than that of coniferous trees, indicating that the drought stress had a greater impact on the NAI release ability of broad-leaved trees. The decline in sap flow of coniferous trees at the end of drought was 77.82 ± 0.65%, and that of broad-leaved trees was 77.65 ± 6.99%; the decline in sap flow of each tree species was greater than that of NAI, indicating that the impact of drought stress on sap flow of each tree species was greater than that on the ability to release NAI.

Studies have shown that after being subjected to drought stress, the ability of each tree species to release NAI and sap flow significantly decreased (*p* < 0.05). Drought stress has a greater impact on the ability of broad-leaved trees to release NAI. Moreover, the impact of drought stress on the sap flow of each tree species is greater than that on NAI.

### 2.2. Changes in NAI and Sap Flow of Different Tree Species During the Recovery Process

**Figure 3:**
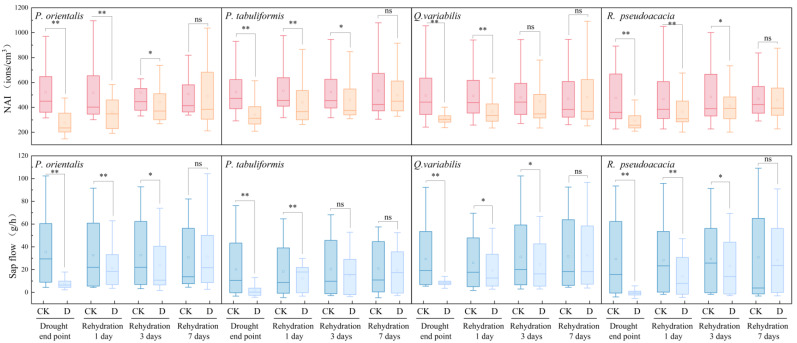
Changes in NAI and sap flow of tree species at rehydration stage. “**, *” indicate significant differences among groups (** *p* < 0.01, * *p* < 0.05), “ns” indicates no statistical difference.

When the plants reach the wilting point, they start to rehydrate, gradually restoring the plant physiology. As the soil moisture recovers, the drought stress is relieved. During the rehydration stage, the NAIC and sap flow of each tree species gradually recover. The characteristics of the rehydration stage are shown in Figure 3.

After 1 day of rehydration, there was still a significant difference between the drought treatment group D and the control group CK (*p* < 0.05). However, the NAIC and sap flow of each tree species in the drought treatment group D recovered significantly. The mean values of NAIC in the control group CK and the drought treatment group D were 473.09 ± 42.15 ions/cm^3^ and 342.53 ± 32.45 ions/cm^3^, respectively. The NAIC of D recovered to 72.41% of that of CK. The mean values of sap flow in CK and D were 32.02 ± 5.47 g/h and 21.59 ± 2.62 g/h, respectively. The sap flow of D recovered to 67.41% of that of CK.

After 3 days of rehydration, except for the drought control group D where the sap flow of *Q. variabilis* and *P. tabulaeformis* was not significantly different from that of the control group CK (*p >* 0.05), the NAIC and sap flow of the other tree species still showed significant differences compared to the control group (*p* < 0.05). After 3 days of rehydration, the mean NAIC of the control group CK and the drought treatment group D were 471.73 ± 40.81 ions/cm^3^ and 405.48 ± 30.62 ions/cm^3^, respectively. The NAIC of D recovered to 85.95% of that of CK. The mean sap flow of CK and D were 34.35 ± 4.52 g/h and 27.02 ± 4.29 g/h, respectively. The sap flow of D recovered to 78.65% of that of CK. The sap flow of broad-leaved trees (79.19% and 81.72%) recovered more than that of coniferous trees (72.52% and 79.13%). The recovery degree of sap flow in broad-leaved trees was better than that in coniferous trees.

After 7 days of rehydration, the NAIC and sap flow of each tree species in the drought treatment group D showed no significant differences compared to the control group CK (*p >* 0.05). Statistically, the NAI and sap flow released by each tree species all returned to the levels of CK. In the drought treatment group D, the NAIC recovery rate of broad-leaved trees was 98.24% compared to the control group. For coniferous trees such as cypress and pine, the NAIC recovery rate was 94.05% of that of the control group. The recovery degree of plant NAI release ability was higher in the broad-leaved tree species than in coniferous tree species.

Studies have shown that after removing the drought stress, the NAIC and sap flow of each tree species in the drought treatment group (D) gradually increased. At 1 day after rehydration, the recovery amplitudes of NAIC and sap flow for each tree species were the greatest; at 3 days after rehydration, the sap flow recovery degree of broad-leaved trees was better than that of coniferous trees; at 7 days after rehydration, statistically, all tree species recovered to the CK level, and the recovery degree of the trees’ NAIC release ability manifested as broad-leaved tree species > coniferous tree species.

### 2.3. Correlation Between NAI Release and Sap Flow by Different Tree Species in the Process of Drought–Rehydration

**Figure 4:**
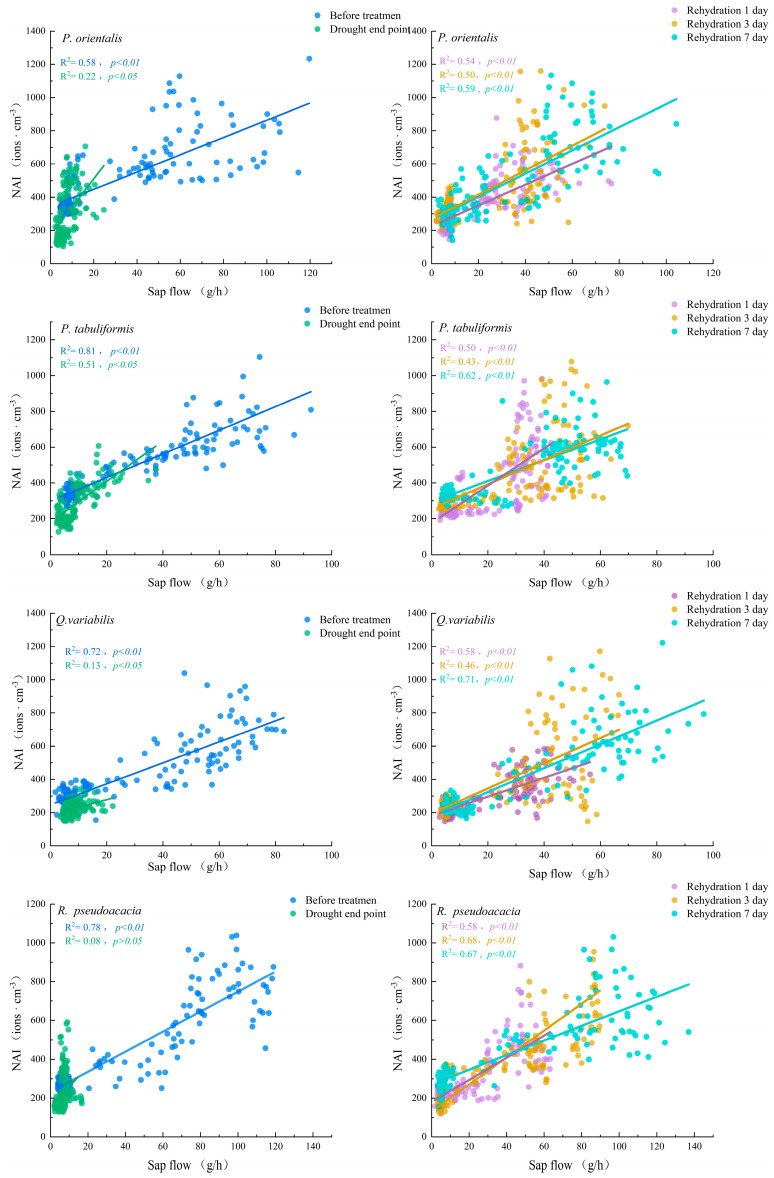
Regression analysis of NAIC and sap flow at stage of drought–rehydration treatment.

Based on the data of each stage during the drought–rehydration process, a correlation analysis and regression analysis were conducted on NAI and sap flow, as shown in Figure 4 and Table 3. The scatter plot and the fitted line visually reflect the correlation characteristics between NAI and sap flow, indicating that for each tree species, NAI was significantly positively correlated with sap flow before the drought treatment (*p* < 0.01).

The R^2^ for each tree species at the drought endpoint was less than that before the drought treatment, and the correlation between NAI and sap flow of honey *R. pseudoacacia* at the drought endpoint became non-correlated (*p* > 0.05), proving that the drought treatment significantly affected the correlation between NAI and sap flow. During the rehydration stage, NAI and sap flow of each tree species were significantly positively correlated (*p* < 0.01); however, there were differences in the correlation for each tree species in the two stages of rehydration for 1 day and 3 days. For example, for Chinese pine (R^2^ = 0.50, *p* < 0.01) and cork oak (R^2^ = 0.58, *p* < 0.01), the correlation between NAI and sap flow was higher at 1 day of rehydration, while for honey locust (R^2^ = 0.68, *p* < 0.01), it was higher at 3 days of rehydration. At 7 days of rehydration, except for *P. orientalis*, the correlation between NAI and sap flow for the other tree species was higher than that before the drought treatment, while the NAI and sap flow correlations for *P. orientalis*, *R. pseudoacacia*, and *Q. variabilis* were lower than those before the drought treatment.

In conclusion, the drought treatment changed the correlation between NAI and sap flow for different tree species. Before the drought treatment, the response degree of broad-leaved trees to NAI was higher than that of coniferous trees, while at the drought endpoint, the response degree of NAI to sap flow was that of coniferous trees > broad-leaved trees, indicating that the drought treatment had a greater impact on the relationship between NAI and sap flow in broad-leaved trees. After 7 days of rehydration, except for *P. orientalis*, the response of NAI to sap flow of the other tree species did not return to its level before the drought treatment.

## 3. Discussion

(1) During the process of drought and rehydration, the NAI values for the green tree species and the trend of sap flow show consistency.

Based on the OTC drought–rehydration experiment, it was found that after drought treatment, the release of NAIC and sap flow of plants were both significantly lower than those of the control group. The recovery amplitude of the release of NAIC and sap flow of plants was the largest on the first day after rehydration. After 7 days of rehydration, the plants in the drought treatment group returned to the CK level. Statistically, there was no significant difference between the drought treatment group and the control group.

Under drought stress, the release of NAIC and sap flow significantly decreased after drought treatment. This might be because drought stress affects the physiological processes of plants and leads to stomatal closure, damage to the photosynthetic system, and a reduction in transpiration rate, among other reactions [19,20]. In this experiment, using potted plants made them more susceptible to hydraulic limitation in the root zone.

During the rehydration stage, after 1 day of rehydration, the recovery amplitude of NAIC and sap flow in the plants was the greatest. This is also in line with current research, which indicates that soil moisture content has a threshold effect on sap flow. When the soil moisture content is too low, it will replace meteorological factors as the main factor influencing plant sap flow. When the soil drought treatment ends and the soil regains saturated moisture content, the restriction of soil moisture content is lifted, and the indicators such as plant sap flow will recover rapidly [21]. Following the drought, the physiological and biochemical changes in the plants, as well as their own protective mechanisms, prevent them from immediately returning to their normal levels [22,23]. And as the drought stress is relieved, the photosynthesis of plants also resumes. Studies have shown that in plant photosynthesis, superoxide NAI O^2−^ produced in photosystem I is one of the main components of NAI [10,24].

After 7 days of rehydration, the sap flow and NAI of the trees basically returned to the levels of the control group (CK), and some tree species showed compensatory effects. Some studies have found that drought stress can cause irreversible damage to plants [25]. However, in this study, such a situation did not occur. On one hand, this might be because the plants used in the experiment were seedlings, which are more sensitive but have stronger recovery ability [26]; on the other hand, it might be because the intensity and duration of the drought were insufficient. In this study, the stress experiment was stopped when the top leaves began to wilt, and rehydration was carried out.

(2) The changes in NAI and sap flow in different tree species vary during the drought–rehydration process.

This study found that at the end of the drought treatment, the decline in NAIC of coniferous trees was less than that of broad-leaved trees, and the decline in sap flow of each tree species was significantly greater than that of NAI. Moreover, the correlation between the response degree of NAI to sap flow was greater for coniferous trees than for broad-leaved trees, indicating that the drought treatment had a greater impact on the relationship between NAI and sap flow in broad-leaved trees. During the rehydration stage, at 7 days after rehydration, the recovery in the NAI release ability of broad-leaved trees exceeded that of coniferous trees.

This study found that the decline in NAIC of coniferous trees was smaller than that of broad-leaved trees. This phenomenon may be related to the morphological characteristics of coniferous trees. From the perspective of leaf morphological structure features, the leaves of coniferous trees are mostly needle-shaped or scale-like. In terms of the number of leaf tips, they have more leaf tips. Current research shows that when the curvature of the leaf is smaller and the number of leaf tips is greater, more NAI will be generated due to corona phenomena [10,27]. Although both coniferous trees and broad-leaved trees were affected by the drought treatment, the NAI of coniferous trees was less affected by the drought treatment due to the corona effect.

This study indicates that the NAI of coniferous trees is significantly less sensitive to the changes in sap flow compared to broad-leaved trees. This phenomenon may be related to the photosynthetic physiological characteristics of the two types of tree species. Relevant studies have shown that when the sap flow rate decreases by 50%, the photochemical efficiency of photosystem II (PSII) in coniferous trees remains above 0.65, while the efficiency of PSII in broad-leaved trees drops sharply to below 0.4. This difference stems from the fact that the photosynthetic apparatus of broad-leaved trees is more sensitive to water stress. Their chloroplast membrane system is more prone to damage under drought conditions, leading to a significant decline in the efficiency of the light reaction, and photosynthesis is an important pathway for plants to produce NAI [28,29]. Meanwhile, the higher basal transpiration rate of broad-leaved trees makes them more prone to water deficit during drought conditions, which in turn leads to the closure of stomata and a comprehensive inhibition of the carbon assimilation process [30].

During the drought treatment period, the decline in sap flow of plants was greater than that of NAI. The reason for this might be related to the NAIC pathway of the plants. Although drought stress causes a decline in physiological processes such as photosynthesis and transpiration in plants, they can still generate NAI through electrical discharge effects at the leaf tips and other parts [31]. The sap flow in plants mainly occurs due to transpiration. When the soil moisture content drops below the wilting coefficient, plants have difficulty absorbing water from the soil, and they will close their stomata to reduce water evaporation from their bodies, thereby making the sap flow even smaller [32,33].

During the rehydration stage, at 7 days of rehydration, the ability of broad-leaved trees to release NAI exceeded that of coniferous trees. The reason for this is that compared to angiosperms, gymnosperms exhibit stronger drought resistance and weaker recovery ability. As coniferous trees, gymnosperms have developed a survival strategy centered on drought tolerance during their long-term evolution. Their xylem vessels have smaller diameters and more compact perforation plate structures, which enhance their ability to resist blockage but also limit the rapid recovery of water transport efficiency [34,35,36]. Therefore, in future studies, it will be possible to combine the structural characteristics of plants, etc., in order to gain a more comprehensive understanding of the mechanisms by which drought affects NAI and sap flow.

## 4. Conclusions

This study used four types of green trees as the experimental materials. Based on an OTC, a control experiment was conducted to monitor the changes in NAI and sap flow at the drought–rehydration stage. It was determined that the drought–rehydration process had a significant impact on the NAI and sap flow of typical green tree species in Beijing. Studies have shown that under drought stress, the NAI and sap flow of each tree species decreased significantly, and the impact on sap flow was greater than that on NAI. After the drought stress was relieved, NAI and sap flow rapidly recovered. After 7 days of rehydration, NAI and sap flow in the drought treatment group returned to the levels of the control group statistically. Furthermore, there were differences in the changes in NAI and sap flow among the different tree species during the drought–rehydration process. During the drought stage, the drought treatment had a greater impact on the NAI release ability of the coniferous trees than that of broad-leaved trees. After 7 days of rehydration, the recovery degree of the plants’ ability to release NAI was found to be greater in broad-leaved tree species than in coniferous tree species. During the drought–rehydration process, the correlation between NAI and sap flow also changed. After the drought treatment, the correlation between NAI and sap flow decreased for all tree species. And after 7 days of rehydration, the correlation between NAI and sap flow for some tree species was even lower than that before the treatment. Of course, the current research selected regional test materials. In future studies, the range of test materials will be expanded, and combined with photosynthetic and transpiration indicators, a more in-depth study will be conducted on the impact of drought on the release of NAI and sap flow by plants.

## Figures and Tables

**Table 1:** Basic growth status of the tree species in this study.

Tree Species	Tree Age (a)	Type of Vegetation	Plant Height (cm)	DBH (mm)
*Pinus tabuliformis*	3	Conifer	86 ± 3.21	11.26 ± 0.86
*Platycladus orientalis*	3	Conifer	120 ± 5.45	12.25 ± 1.24
*Robinia pseudoacacia*	3	Broad-leaved tree	130 ± 8.67	10.21 ± 0.22
*Quercus variabilis*	3	Broad-leaved tree	91 ± 4.91	8.25 ± 0.52

Note: DBH, diameter at breast height.

**Table 2:** Basic properties of soil.

Soil Bulk Density (g/cm^3^)	Total Nitrogen (g/kg)	Total Phosphorus (g/kg)	Total Potassium (g/kg)	Field Capacity (g/kg)
1.27	1.108	1.132	19.73	357.41

**Table 3:** The correlations between NAI released by different tree species and sap flow during drought and rehydration.

Tree Species	Before Treatment	Drought Endpoint	Rehydration 1 Day	Rehydration 3 Days	Rehydration 7 Days
*Platycladus orientalis*	0.762 **	0.469 *	0.739 **	0.706 **	0.768 **
*Pinus tabuliformis*	0.843 **	0.451 *	0.707 **	0.656 **	0.787 **
*Robinia pseudoacacia*	0.883 **	0.294	0.761 **	0.824 *	0.818 **
*Quercus variabilis*	0.849 **	0.360 *	0.760 **	0.678 **	0.842 **

** *p* < 0.01, * *p* < 0.05 (two-tailed).

## Data Availability

The data that support the findings of this study are available on request from the corresponding author.
